# Microcirculatory tissue oxygenation correlates with kidney function after transcatheter aortic valve implantation–Results from a prospective observational study

**DOI:** 10.3389/fcvm.2023.1108256

**Published:** 2023-02-14

**Authors:** Maximilian Dietrich, Ana Antonovici, Tobias Hölle, Christian Nusshag, Anne-Christine Kapp, Alexander Studier-Fischer, Rawa Arif, Felix Nickel, Markus Alexander Weigand, Norbert Frey, Christoph Lichtenstern, Florian Leuschner, Dania Fischer

**Affiliations:** ^1^Department of Anesthesiology, Heidelberg University Hospital, Heidelberg, Germany; ^2^Department of Nephrology, Heidelberg University Hospital, Heidelberg, Germany; ^3^Department of General, Visceral and Transplantation Surgery, Heidelberg University Hospital, Heidelberg, Germany; ^4^Institute of Cardiac Surgery, Heidelberg University Hospital, Heidelberg, Germany; ^5^Department of Internal Medicine III, Heidelberg University Hospital, Heidelberg, Germany

**Keywords:** risk assessment, monitoring, TAVR-transcatheter aortic valve replacement, microcirculation, hyperspectral imaging (HSI), kidney function, cardiovascular interventions, TAVI–transcatheter aortic valve implantation

## Abstract

**Introduction:**

Kidney dysfunction is common in patients with aortic stenosis (AS) and correction of the aortic valve by transcatheter aortic valve implantation (TAVI) often affects kidney function. This may be due to microcirculatory changes.

**Methods:**

We evaluated skin microcirculation with a hyperspectral imaging (HSI) system, and compared tissue oxygenation (StO_2_), near-infrared perfusion index (NIR), tissue hemoglobin index (THI) and tissue water index (TWI) in 40 patients undergoing TAVI versus 20 control patients. HSI parameters were measured before TAVI (t1), directly after TAVI (t2), and on postinterventional day 3 (t3). The primary outcome was the correlation of tissue oxygenation (StO_2_) to the creatinine level after TAVI.

**Results:**

We performed 116 HSI image recordings in patients undergoing TAVI for the treatment of severe aortic stenosis and 20 HSI image recordings in control patients. Patients with AS had a lower THI at the palm (*p* = 0.034) and a higher TWI at the fingertips (*p* = 0.003) in comparison to control patients. TAVI led to an increase of TWI, but had no uniform enduring effect on StO_2_ and THI. Tissue oxygenation StO_2_ at both measurement sites correlated negatively with creatinine levels after TAVI at t2 (palm: ρ = −0.415; *p* = 0.009; fingertip: ρ = −0.519; *p* < 0.001) and t3 (palm: ρ = −0.427; *p* = 0.008; fingertip: ρ = −0.398; *p* = 0.013). Patients with higher THI at t3 reported higher physical capacity and general health scores 120 days after TAVI.

**Conclusion:**

HSI is a promising technique for periinterventional monitoring of tissue oxygenation and microcirculatory perfusion quality, which are related to kidney function, physical capacity, and clinical outcomes after TAVI.

**Clinical trial registration:**

https://drks.de/search/de/trial, identifier DRKS00024765.

## Introduction

Kidney dysfunction is common in patients with aortic stenosis and both entities bilaterally influence each other (1). Several studies have shown that post-procedure damage to the kidneys, acute kidney injury (AKI), is associated with worse clinical outcomes after transcatheter aortic valve implantation (TAVI) (2, 3). Depending on the definition used, AKI occurs in 3.4–57% of TAVI patients after the intervention (2). Even small decreases in kidney function can have a dramatic impact on the risk of subsequent mortality, which is not unique to TAVI, as impairment of kidney function is consistently associated with worse clinical outcomes (4).

On the other side, kidney function improves in many patients after TAVI, presumably due to enhanced hemodynamics including tissue and organ perfusion (5). We postulate that there might be potential clinical applicability of microcirculatory hyperspectral imaging for screening and risk stratification of post-TAVI changes in kidney function (6). Hyperspectral imaging is an innovative diagnostic tool to visualize hemodynamic changes by showing the oxygen saturation of hemoglobin in the capillary system, the distribution of hemoglobin in the tissue and the relative tissue water content. In a previous porcine model study, we used hyperspectral imaging to investigate the microcirculation of the skin and kidney during hemorrhagic shock and resuscitation. The tissue oxygenation parameters of the skin and kidneys correlated, and we additionally found a correlation to established indirect markers of tissue oxygenation (7).

If early changes in tissue oxygenation and perfusion in response to TAVI prove to be outcome-associated, this technical method enabling bedside microcirculatory monitoring may be a highly valuable tool for risk-stratification. Our previous work in septic patients showed lower tissue oxygenation and an increased water content of the patients’ skin compared to healthy volunteers (8). Furthermore, tissue hemoglobin content of the palm and the fingertip on admission to the intensive care unit was lower in 28 day-survivors and had a high predictive value for 28-day mortality. Another study in patients undergoing pancreatic surgery showed that skin tissue oxygenation and hemoglobin content of the palm increased in response to anesthetic induction (9). Tissue water content increased during surgery and was related to the duration of surgery, intraoperative fluid balance and blood loss, as well as the change of the syndecan-1 levels as a biomarker of glycocalyx damage. Hence, our previous work shows that hyperspectral imaging may be a valuable tool to bridge a diagnostic gap that might be useful to tailor individualized nephroprotective patient care.

The present study aims to investigate whether and how changes in microcirculation after TAVI can be detected with hyperspectral imaging and whether microcirculatory perfusion quality is associated with kidney function, physical capacity, and clinical outcomes.

## Materials and methods

### Study design and settings

This prospective, observational pilot study was conducted by the Department of Anesthesiology, the Department of Cardiology, Angiology and Pneumology, and the Department of Cardiac Surgery of Heidelberg University Hospital, Germany. The local Institutional Ethics Committee of the Medical Faculty of Heidelberg University approved the study (reference number S-128/2021). The study has been performed in accordance with the ethical standards laid down in the 1964 Declaration of Helsinki and its later amendments. The study was registered at the German Clinical Trial Register (DRKS00024765). The reporting of the study adheres to the STROBE guidelines (10).

### Participants

Forty patients with severe aortic stenosis undergoing TAVI were recruited on hospital admission and informed consent to participate was obtained. Inclusion criteria of the TAVI group were age ≥18 years, signed informed consent, elective transcatheter aortic valve implantation for the therapy of an aortic stenosis. Exclusion criteria were transcatheter aortic valve implantation for the treatment of aortic regurgitation, refusal of participation, infectious viral diseases [Hepatitis B virus (HBV), Hepatitis C virus (HCV), HIV, COVID-19] or unknown COVID-19 status, chronic kidney failure with a glomerular filtration rate (GFR) < 15 ml/min/m^2^ or need for dialysis. As a control group, we recruited 20 adult patients hospitalized ahead of coronary artery bypass graft (CABG) surgery. Informed consent to participate was obtained from all control group patients. Exclusion criteria for the control group were aortic valve pathology, moderately or highly impaired left ventricular function and chronic kidney failure with a glomerular filtration rate (GFR) < 15 ml/min/m^2^ or need for dialysis.

### Hyperspectral imaging

Hyperspectral imaging technique for the non-invasive, bedside measurement of tissue oxygenation, hemoglobin and water content is based on the specific light reflection and absorption of different compounds such as oxy-/deoxyhemoglobin and water. The TIVITA^®^ Tissue System (Diaspective Vision GmbH, Am Salzhaff, Germany), a CE-certified and validated class I medical device, was used for HSI measurements. The camera system was applied following the operating instructions and European medical device regulation. The physical principles of the measurement, technical specifications, and algorithms of the HSI camera system have been described in detail by Holmer et al. (11). A measurement procedure requires only a few seconds and allows the calculation of the following parameters from specified wavelength ranges (6, 11):

- Tissue oxygenation (StO_2_, wavelength range: 500–650 and 700–815 nm): oxygen saturation of the hemoglobin in the superficial capillary system (penetration depth up to 1 mm);
- NIR perfusion index (NIR, wavelength range: 655–735 and 825–925 nm): oxygen saturation of the hemoglobin in the capillary system of deeper tissue layers (penetration depth up to 4–6 mm);
- Tissue hemoglobin index (THI, wavelength range: 530–590 and 785–825 nm): distribution of deoxygenated and oxygenated hemoglobin in the measured tissue (penetration depth up to 1–3 mm);
- Tissue water index (TWI, wavelength range: 880–900 and 955–980 nm): relative tissue water content (penetration depth up to 1–3 mm).

**FIGURE 1:**
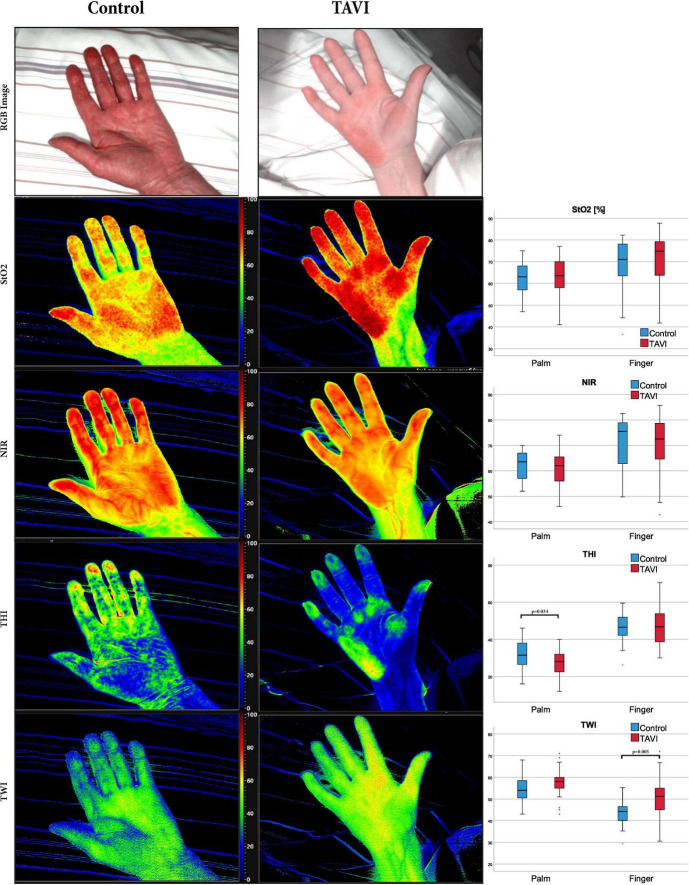
Red-green-blue image and images derived from HSI of the palm. Boxplots show the respective HSI measurements of TAVI and Control patients; Tissue oxygenation (StO2), near-infrared perfusion index (NIR), tissue hemoglobin index (THI) and tissue water index (TWI) are displayed color-coded (scale at the right side of each image). Red/yellow areas indicate high values (50–100), green/blue (0–50) areas indicate low values. The numerical scale ranges from 0 to 100. StO2 is given in% and THI, NPI, and TWI are index values in arbitrary units. Mann–Whitney-U test was used for comparisons between groups. HSI, hyperspectral imaging; CABG, coronary artery bypass graft; TAVI, transcatheter aortic valve implantation. Additional numerical values are shown in Supplementary Table 1.

Tissue oxygenation is indicated in percent (0–100%), NIR, THI, and TWI are indicated in predefined arbitrary units (0–100). The parameters are displayed to the operator as color-coded images in addition to the RGB-image (Figure 1). Areas with high values (50–100) are shown in shades from red to yellow, whereas areas with low values (0–50) are presented in green to blue shades. HSI measurements were performed on the right hand, using the analysis software circular regions of interest with a diameter of 70 units were defined in palm area (the circle crossing the metacarpophalangeal joint 3–4) while the diameter used for the fingertips 2–5 measured only 13 units. The mean of the four values was therefrom calculated. The treating physicians had no access to the HSI measurement results to avoid an influence on clinical therapy.

### Anesthesia, hemodynamic management, and aortic valve replacement

All implantation procedures were performed in analgosedation using propofol and remifentanil in addition to local anesthesia. The treating anesthesiologist determined the hemodynamic therapy during the procedure. A multidisciplinary cardiac team set the indication for aortic valve replacement and the type of valve implantation. Access route, device type and size, as well as all the specific implantation proceedings were determined by the attending interventional cardiologist. Afterward, the patients were admitted to the intensive or intermediate care unit of the Department of Cardiology, Angiology and Pneumology.

### Measurements

In patients undergoing aortic valve replacement, HSI parameters were measured before the aortic valve implantation (t1), directly after the aortic valve implantation procedure (t2), and on the third postinterventional day (t3). Blood samples and hemodynamic parameters were collected, respectively. Blood samples from central and arterial lines for the measurement of the pCO_2_ gap and central venous oxygen saturation (ScvO_2_) could only be collected at t1 and t2 due to early removal of the catheters. Demographic data, comorbidity, medication and data concerning the procedure, blood loss, administered fluids, catecholamines, and anesthetics were documented. To evaluate endothelial glycocalyx integrity, serum syndecan-1 was measured by ELISA (R&D Systems). Echocardiography, performed by a cardiologist, was used to determine the preinterventional aortic valve opening area and the pre- and postinterventional ejection fraction. To evaluate the postinterventional survival and physical functioning, the patients were contacted by telephone 120 days after TAVI. To assess physical capacity, we used the scales Physical Functioning, Energy/Fatigue, Role Limitations due to Physical Health, and General Health of the Short Form-36 survey (12, 13). Each item is scored from 0 to 100, with high values representing the favorable health condition. The scales are calculated as the mean of the respective items.

### Primary outcome

The primary outcome was the tissue oxygenation parameter StO_2_ on the hand and fingertips after transcatheter aortic valve implantation and its correlation to the creatinine level as a marker of kidney function.

### Secondary outcome

Secondary endpoints were the course of HSI parameters NIR, THI and TWI and their comparison between patients with aortic stenosis to the control group. Further the patient cohort was divided based on the median StO2, THI and TWI at t3 into groups higher and equal versus lower than the median. The resultant groups were compared considering kidney function, SF-36 physical capacity and health scores, as well as the length of ICU and hospital stay.

### Statistical methods

Data was collected with the aid of an electronic database system (Microsoft Excel^®^, Microsoft Deutschland GmbH, Unterschleißheim, Germany). SPSS (Statistical Product and Services Solutions, Version 28, SPSS Inc., Chicago, IL, USA) was used for statistical analyses. Descriptive statistics were done for the complete dataset. For continuous variables and scores, mean, standard deviation, minimum, median, quartiles and maximum were calculated and median values with interquartile range (IQR) are presented in the manuscript. Absolute and relative frequencies of categorical variables were presented. Spearman rank correlation was applied to values of HSI parameter with hemodynamic parameters and kidney function parameters. A Friedman test was used to analyze changes over time. A Wilcoxon test was used for the comparison of metric data between paired samples. A Mann–Whitney U test was used for the comparison of metric data between unpaired samples. Results of the statistical tests must be considered descriptive.

## Results

Forty patients undergoing TAVI for the therapy of a severe aortic stenosis and 20 control patients without aortic valve pathology hospitalized ahead of CABG were included. Patient characteristics, comorbidities, medication, and the used valve devices are shown in Table 1. All patients of the TAVI group had an aortic valve opening area ≤ 1 cm^2^. Median duration of the TAVI procedure was 35 min (IQR 35; 40). Nine (22.5%) patients required a pacemaker after the intervention because of higher-grade atrioventricular block. The median stay on the intensive care unit was 2 days (IQR 2;2) and the median length of hospital stay was 13 days (IQR 8;19). We were able to follow-up 35 patients after 120 days and achieved completion of the SF-36 in 28 patients. The 30- and 120-day mortality was 2.6% (1/39) and 8.6% (3/35).

**TABLE 1:** Patient Characteristics; categorical values are given in absolute and relative frequencies, a Chi-Squared-Test was used for analysis.

		TAVI	Control	*p*
Age		82 (78;85)	71 (67;77)	<0.001
Sex (male)		21 (52.5%)	14 (70%)	0.195
BMI		25.6 (23.2;30.7)	27 (24;31.5)	0.515
Bodysurface [m^2^]		1.9 (1.74;2.03)	2 (1.77;2.12)	0.24
Creatinine		0.95 (0.71;1.13)	1.01 (0,76; 1.42)	0.393
ASA	III	17 (42.5%)	13 (68.4%)	0.063
	IV	23 (57.5%)	6 (31.6%)	
NYHA	I	0 (0%)	2 (12.5%)	0.015
	II	8 (20%)	7 (43.75%)	
	III	27 (67.5%)	7 (43.75%)	
	IV	5 (12.5%)	0 (0%)	
Euroscore		7 (6;9)	5 (3;7)	0.005
**Preexisting medical conditions**				
Adipositas		14 (35%)	9 (45%)	0.453
Hypertension		40 (100%)	14 (73.7%)	<0.001
IDDM		17 (42.5%)	10 (52.6%)	0.465
COPD		13 (33.3%)	5 (25%)	0.511
CKD		15 (39.5%)	2 (10%)	0.019
Myocardial infarction		5 (12.5%)	4 (20%)	0.443
Atrial fibrillation		19 (47.5%)	2 (10%)	0.004
Stroke		2 (5%)	0 (0%)	0.309
Peripheral vessel disease		7 (17.5%)	3 (15%)	0.806
Tabac use	Active	4 (10%)	4 (20%)	0.544
	Abstinent	10 (25%)	5 (25%)	
Hypercholesterolemia		25 (65.8%)	14 (73.7%)	0.546
**Medication**				
Aspirin		18 (45%)	16 (80%)	0.01
Beta-blocker		31 (77.5%)	11 (55%)	0.073
Statins		28 (70%)	17 (85%)	0.206
Diuretics		28 (70%)	7 (35%)	0.01
ACE/ARBs		33 (82.5%)	10 (50%)	0.008
Antimineralocorticoids		6 (15%)	3 (15%)	1
**Intervention**				
Intervention duration [min]		35 (35;40)		
Peri interventional fluid [ml]		700 (500;1100)		
Contrast agent [ml]		53 (40;85)		
ICU stay [d]		2 (2;2)		
Hospital stay [d]		13 (8;19)		
Ejection fraction [%]	Pre intervention	55 (40;58)	56 (52;60)	0.14
	Post intervention	55 (42;60)		
Aortic valve area [cm^2^]		0.775 (0.7;0.85)		
**Mean aortic valve pressure gradient**				
	Pre intervention	35 (26;41)		
	Post intervention	4 (4;6)		
Valve	2 × Evolut 26 mm*	1 (2.6%)		
	Edwards 23 mm	1 (2.6%)		
	Edwards 29 mm	3 (7.7%)		
	Edwards Sapien 26 mm	1 (2.6%)		
	Evolut 22 mm	1 (2.6%)		
	Evolut 26 mm	16 (41%)		
	Evolut 29 mm	8 (20.5%)		
	Evolut 29 mm Pro	2 (5.1%)		
	Evolut 34 mm	6 (15.4%)		

Continuous values are reported as mean and interquartile range, a Mann–Whitney-U test was used for comparisons.*The patient received a second valve in the same session due to non-ideal placement of the first valve. BMI, body mass index; ASA, American Society of Anesthesiologist Physical Status Classification System; NYHA, New York Heart Association Functional Classification; IDDM, insulin dependent diabetes mellitus; COPD, chronic obstructive pulmonary disease; CKD, chronic kidney disease; ACE, angiotensin-converting enzyme inhibitors; ARB, angiotensin receptor blockers; ICU, intensive care unit.

### Patients with aortic stenosis had a lower tissue hemoglobin and a higher tissue water content in comparison to control patients

We performed 116 HSI image recordings with measurements at the palm and the fingertips in patients undergoing TAVI for the treatment of aortic stenosis and 20 HSI image recordings in control patients. Exemplary hyperspectral images of one patient before TAVI and one patient of the control group are shown in Figure 1. We compared the HSI parameters before TAVI (t1: 39 image recordings) with the measurements of the control group to evaluate the effects of an aortic stenosis on tissue oxygenation, hemoglobin and water content (Figure 1). Both groups were measured at rest under stable macrohemodynamic conditions. Mean arterial pressure and heart rate did not differ significantly between TAVI and control patients. Patients with aortic stenosis had a lower THI at the palm (*p* = 0.034), whereas a higher TWI was observed at the fingertips (*p* = 0.003). The same trend toward a higher TWI was observed at the palm (*p* = 0.052).

### Transcatheter aortic valve implantation increased tissue water content, but had no uniform enduring effect on tissue oxygenation and tissue hemoglobin content

**FIGURE 2:**
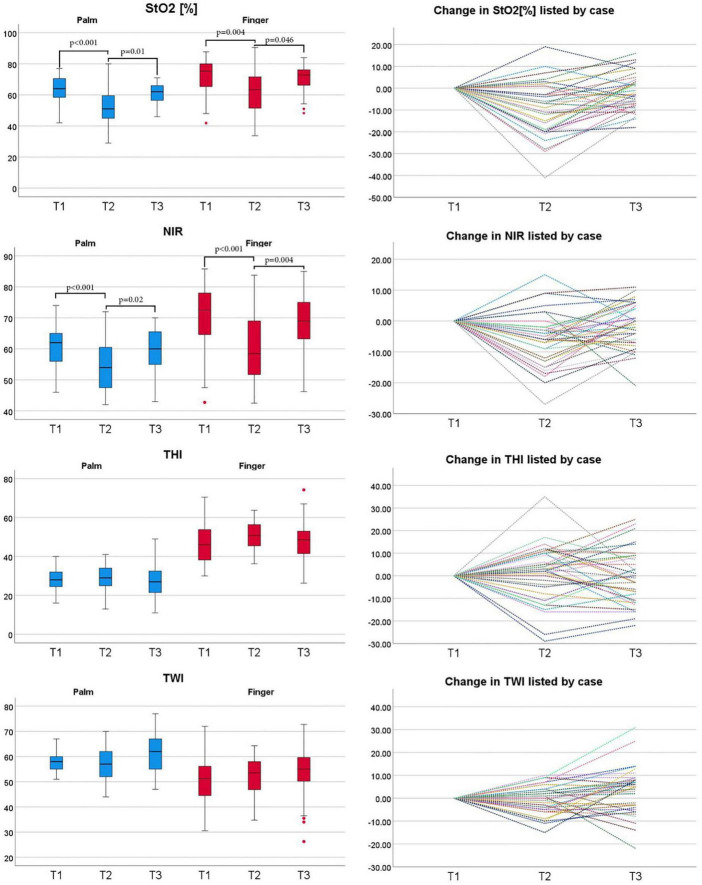
The course of parameters derived from HSI in TAVI patients at the palm (blue) and fingertips (red). Line graphs show individual course of HSI measurements over time; HSI parameters were taken at three different timepoints: before valve replacement (T1), after valve replacement (T2) and 3 days after valve replacement (T3). Tissue oxygenation (StO2), tissue hemoglobin index (THI), near-infrared perfusion index (NPI), tissue water index (TWI) values range from 0 to 100. StO2 is given in% and THI, NPI, and TWI are index values in arbitrary units. Friedman-test was used for the statistical analysis. HSI, hyperspectral imaging; CABG, coronary artery bypass graft; TAVI, transcatheter aortic valve implantation.

**FIGURE 3:**
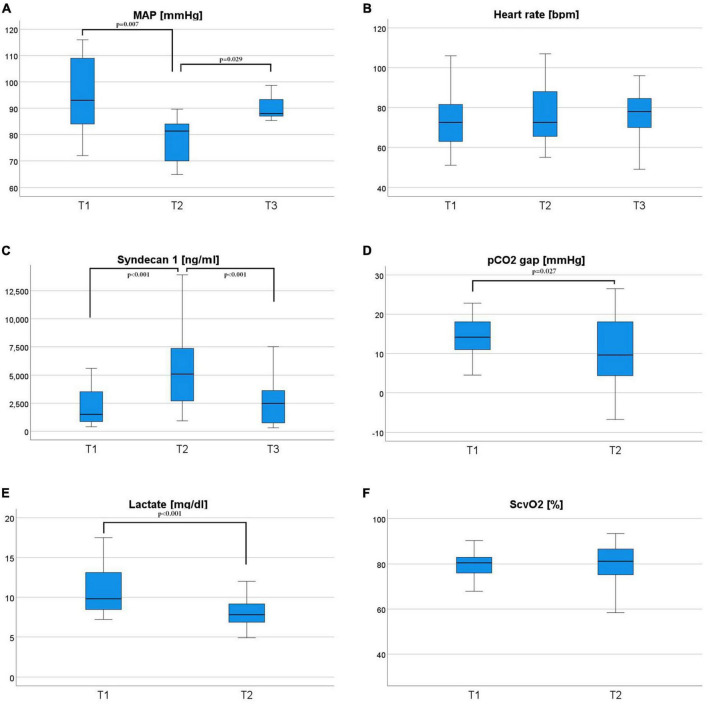
The course of hemodynamic parameters over time; Mean arterial pressure (a MAP) **(A)** is given in millimeters of mercury (mmHg), heart rate **(B)** is given in beats per minute (bpm), Syndecan-1 **(C)** is given in ng/ml, carbon dioxide gap (pCO2 gap) **(D)** is given in millimeters of mercury (mmHg), lactate **(E)** is given in milligrams per deciliter (mg/dl) and the central venous blood oxygenation is given in percent. Parameters were measured at two or three timepoints: before valve replacement (T1), after valve replacement (T2), and **(F)** 3 days after valve replacement (T3) for MAP, HR, and Syndecan-1. Friedman-test was used for the statistical analysis.

Boxplots and the patient’s individual courses of HSI parameters of tissue oxygenation, hemoglobin, and water content are presented in Figure 2. Tissue oxygenation StO_2_ and NIR decreased from the measurement before the TAVI procedure to the measurement directly after valve implantation in both the palm and the fingertips. On the third postinterventional day there was no significant difference in StO_2_ or NIR compared to the measurement before valve implantation. THI did not significantly change during the periinterventional period. TWI of the palm increased continuously from t1 to t2 and t2 to t3. There was no significant change of the TWI at the fingertip. The course of macrohemodynamics, established markers for tissue perfusion, and the glycocalyx biomarker Syndecan-1 are shown in Figure 3.

### Tissue oxygenation after valve replacement correlated negatively with kidney function

**FIGURE 4:**
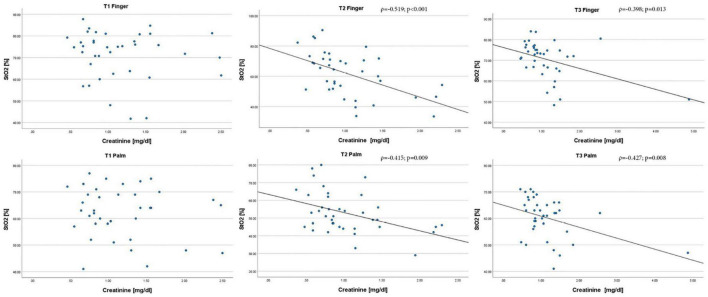
Scatterplots of the correlation analysis of S_t_O_2_ derived from HSI with creatinine levels at different timepoints and measurement sites; HSI parameters and creatinine levels were taken at three different timepoints: before valve replacement (T1), after valve replacement (T2) and 3 days after valve replacement (T3). Creatinine levels are shown on the x-axis and the corresponding S_t_O_2_ values are shown on the y-axis. S_t_O_2_ is given in%, creatinine is given in mg/dl. Spearman correlation was used. S_t_O_2_, tissue oxygenation; ρ, coefficient of correlation.

The median StO_2_ of the palm on the third postinterventional day was 62% (56;66). We compared patients with higher to lower tissue oxygenation of the palm at t3. Patients with higher tissue oxygenation of the palm at t3 had a significantly lower creatinine level at t3 [higher vs. lower StO_2_ t3: 0.84 (0.67;1.06) vs. 1.25 (0.82;1.48) mg/dl; *p* = 0.05]. Tissue oxygenation StO_2_ at both measurement sites correlated negatively with creatinine levels after aortic valve replacement at t2 (palm: ρ = −0.415; *p* = 0.009; fingertip: ρ = −0.519; *p* < 0.001) and t3 (palm: ρ = −0.427; *p* = 0.008; fingertip: ρ = −0.398; *p* = 0.013). The scatterplots of the correlation analyses of StO_2_ with creatinine levels are shown in Figure 4. Physical capacity and general health scores in the assessment after 120 days did not differ between patients with higher or lower StO_2_ of the palm at t3.

### Higher tissue hemoglobin content after valve replacement was associated with higher physical capacity and general health

**FIGURE 5:**
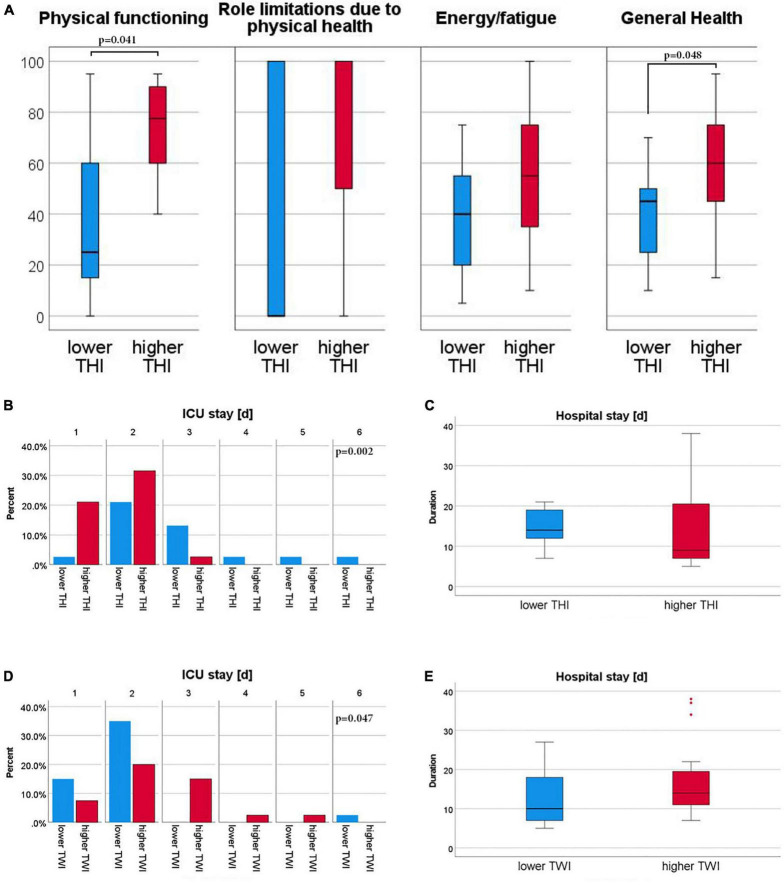
Subgroup analysis for THI and SF-36 as well as subgroup analysis of THI and TWI groups for hospital and ICU stay; Patients were divided by median values of Tissue Hemoglobin Index (THI) and Tissue Water Index (TWI), respectively into a higher than or equal the median (red) and lower than median (blue) group. A total of 36-Item Short Form Health Survey (SF-36) individual scores are shown for the THI groups **(A)**. All Scores are given on an arbitrary scale of 0–100, high values representing the favorable health condition. Stay on Intensive Care Unit (ICU) is given by relative frequency for THI **(B)** and TWI **(D)** groups by days [d]. Hospital stay is given for THI **(C)** and TWI **(E)** subgroups in days [d]. Mann–Whitney-U test was used for comparisons between groups.

The median THI of the palm on the third postinterventional day was 25 (21;32). We compared patients with higher to lower THI of the palm at t3. Patients with higher THI had a higher physical functioning score [higher vs. lower THI t3: 75 (55:90) vs. 23 (10:60); *p* = 0.041] and a non-significant trend toward fewer role limitations due to physical health [higher vs. lower THI t3: 88 (50;100) vs. 0 (0;100); *p* = 0.077]. Further patients with higher THI achieved higher scores in general health [higher vs. lower THI t3: 60 (45:75) vs. 45 (25:50); *p* = 0.048]. The comparison physical capacity in patients with higher or lower tissue hemoglobin content is shown in Figure 5.

### Higher tissue water and lower tissue hemoglobin content indicated a protracted recovery after TAVI

The median TWI of the palm on the third postinterventional day was 62.0 (55;67). We compared patients with higher to lower tissue water, as well as higher to lower tissue hemoglobin content of the palm at t3. Both in patients with higher TWI and in patients with lower THI on the third day after TAVI, a longer ICU-stay and a non-significant trend toward a longer hospital stay was observed (Figure 5). TWI did not correlate with the levels of the glycocalyx biomarker Syndecan-1 and the periinterventional fluid balance.

## Discussion

Morbidity and mortality after TAVI have decreased in the last decade as both the procedure and the transcatheter aortic valve prostheses have greatly improved (14–16). Enhanced risk prediction models are under continuous development and have great potential to further improve risk management by pathing the way for individualized patient care (17). AKI remains a relevant endpoint after TAVI and nephroprotective strategies could be applied for those identified to be at risk. These strategies include euvolemia at the time of TAVI, contrast-sparing techniques, discontinuation of concomitant nephrotoxins such as aminoglycosides, non-steroidal anti-inflammatory agents, or vancomycin (18).

From Perlman et al. (1), it is known that positive response to TAVI in macrohemodynamic parameters is associated with favorable long-term outcome. We hypothesized that this is due to improvement in organ tissue perfusion and oxygen delivery, assuming a hemodynamic coherence of the macro- and microcirculation. Based on this assumption, we postulated that microcirculatory assessment may also develop into a valuable risk-prediction tool. However, up until now, a direct monitoring of microcirculatory response to TAVI is not established in clinical practice. Hyperspectral Imaging is an innovative, non-invasive technology that allows an objective evaluation of tissue oxygenation, perfusion quality, and tissue water content based on specific light reflection and absorption characteristics of substances like oxy-/deoxyhemoglobin or water (19). As AKI is still a relevant complication after TAVI, the primary endpoint of this study was the correlation of microcirculatory imaging parameters with kidney function represented by serum creatinine.

In this first study assessing the microcirculatory response to TAVI in patients with severe aortic stenosis, we used the TIVITA^®^ Tissue System to measure Tissue oxygenation (StO_2_), Near-Infrared Perfusion Index (NIR), Tissue Hemoglobin Index (THI), and Tissue Water Index (TWI) before, directly after and on day 3 after TAVI. With the aim to assess the effect of an aortic stenosis on the microcirculation we compared the measurements in patients before TAVI to a control group of patients before coronary artery bypass grafting, who were known to have a healthy aortic valve and a good ventricular function.

The clinical examination of the skin microcirculation using capillary refill time, skin temperature and color, as well as the search for skin mottling is an integral part of any comprehensive hemodynamic assessment. The skin is easily accessible for measurements and an association of skin microcirculatory alterations to organ failure and mortality has been shown in different cohorts (20–22). In a previous study we evaluated different measurement sites for hyperspectral imaging: the patient’s palm, fingertips, and knee including the front of the thigh. As the palm and the fingertips turned out to be advantageous over the knee measurement site, we decided to use these measurement sites in the present study (8).

In comparison to control patients scheduled for CABG, patients with aortic stenosis revealed a lower tissue hemoglobin and a higher tissue water. The tissue oxygenation parameters StO_2_ and NIR did not differ significantly. Both cohorts were measured at rest under stable macrohemodynamic conditions. Lower perfusion due to aortic stenosis could be a reason for the decreased tissue hemoglobin content. The lack of difference in tissue oxygenation could be explained through sufficient cardiac output in patients with aortic stenosis ensuring adequate tissue oxygenation and that critical tissue perfusion may only become apparent in stress situations. This hypothesis is reinforced by the observation that the patients before TAVI were mostly symptom-free at rest. In line, established markers of tissue oxygenation such as lactate and macrohemodynamic parameters did not show any difference between the two groups. However, TAVI patients had a worse renal function concerning glomerular filtration rate and a higher proportion of patients had a history of chronic kidney disease, possibly leading to the pronounced tissue water content of the TAVI cohort.

Considering the median course of HSI parameters, TAVI had no uniform enduring effect on tissue oxygenation and tissue hemoglobin content. We observed reduced tissue oxygenation parameters directly after TAVI, but StO_2_ and NIR returned to the initial level before TAVI until postinterventional day 3. The visualization of the HSI parameters and their development individually for each patient, revealed a heterogeneous response to TAVI. This suggests an individual effect on the microcirculation where HSI bears the potential to deliver additional diagnostic and prognostic information for personalized treatment.

The primary endpoint of this study was the correlation of the tissue oxygenation parameter StO_2_ to kidney function represented by serum creatinine. Tissue oxygenation StO_2_ both at the palm and the fingertip correlated negatively with creatinine levels directly after aortic valve replacement and on postinterventional day 3. In line with these findings, tissue oxygenation of the skin and the kidney correlated in a porcine model of hemorrhagic shock (7). Since adequate tissue oxygenation depends on sufficient perfusion with oxygenated blood, this indicates a relevant association between skin and organ perfusion. We were not able to assess the relation between the periinterventional changes of skin oxygenation and acute kidney injury, as only one patient developed postprocedural AKI. In this patient, StO_2_ and NIR of the palm and fingertip worsened drastically after TAVI. To assess the link between organ failure and microcirculation measured by HSI, further studies in critically ill patients, e.g., with refractory shock or on veno-arterial Extracorporeal membrane oxygenation (ECMO) for circulatory support are required. The phenomenon of hemodynamic incoherence between macro- and microcirculatory parameters in these patients justifies the necessity for bedside tissue perfusion monitoring.

Tissue oxygenation on postinterventional day 3 was not predictive for physical capacity and general health scores in the assessment after 120 days. This matches before mentioned results of the comparison of tissue oxygenation of patients with aortic stenosis to control patients. At rest, cardiac output in asymptomatic patients seems sufficient to maintain tissue oxygenation. StO_2_ at rest may not predict the cardiocirculatory capacity to perform activities with higher physical intensity. In future studies, HSI measurements during physical activity might provide further insights.

Tissue hemoglobin index measures the total distribution of both oxygenated and deoxygenated hemoglobin in the tissue. A higher THI on the third day after TAVI was associated with higher physical capacity and general health scores. Furthermore, a higher THI was associated with faster recovery regarding hospital and ICU stay. In a study of Thiem et al. (23). HSI was used for the perioperative monitoring of transplanted flaps: an increase of THI in combination with low oxygenation was a sign for venous congestion of the flap. In sepsis patients a high THI on admission to the intensive care unit had a predictive value for mortality (8). In a porcine model of hemorrhagic shock, norepinephrine administration without adequate fluid resuscitation led to an ascent of THI at the skin and a decrease of THI at the kidney (7). Considering the results of both studies, an increase in THI can probably have various causes and must be interpreted in conjunction with tissue oxygenation and in light of the underlying pathophysiology. In sepsis, we proposed that an increased THI could be a sign of red blood cell pooling in the skin capillaries as a consequence of a stagnant flow situation. Excessive norepinephrine administration without adequate fluid resuscitation could potentially amplify this effect by vasoconstriction of the inflow and especially outflow vessels. As a counter-mechanism to congestion, an increase in tissue hemoglobin content through an increase in perfusion is conceivable. Combining the parameters of tissue oxygenation and the patient’s clinical condition could be useful to distinguish between the two. In case of stagnant capillary flow, low oxygenation would be expected according to the observations in venous congestion of transplanted flaps. In case of simultaneous adequate oxygenation, one would rather assume an increase in perfusion.

Tissue water index changes could be driven by periinterventional fluid administration or capillary leakage with fluid extravasation. Fluid overload is associated with worse clinical outcomes and mortality (24, 25). So far there is no available technical method for the quantitative assessment of tissue water content. In a previous study we observed a perioperative increase of TWI in patients undergoing pancreatic surgery, which was more pronounced due to longer duration of surgery, higher intraoperative fluid balance, and blood loss (9). Patients with sepsis had a significantly higher TWI than healthy volunteers, possibly as a sign of sepsis induced capillary leakage (8). In a study on HSI for wound evaluation a decrease in TWI indicated a reduction of wound edema (26). An experimental study showed that fluid resuscitation was accompanied by an increase in TWI, which correlated strongly with the cumulative amount of administered fluid (7). Tissue water content, as well as Syndecan-1, a marker of glycocalyx integrity, increased after TAVI. In patients undergoing pancreatic surgery, Syndecan-1 correlated with TWI kinetics. Yet in TAVI patients there was no plausible correlation of Syndecan-1 or periinterventional fluid balance and TWI, pointing to an influence of multiple factors other than endothelial damage on tissue water content. However, patients with higher TWI on the third day after valve replacement had a longer ICU-stay and a non-significant trend toward a longer hospital stay. This suggests that measurement of tissue water content by TWI could be a useful extension of current hemodynamic monitoring for risk stratification after TAVI.

Several limitations must be recognized for a conclusive interpretation of the results of the present study. First, this was a small pilot study to explore hyperspectral imaging for the evaluation of microcirculatory effects in patients undergoing TAVI. Follow-up studies with larger numbers of patients are needed to confirm our findings and hypotheses. Further, we were not able to report the SF-36 for all patients. However, we assessed health-associated quality of life including physical capacity after 120 days in 70% of patients with high age and disease burden. We could not investigate the relation of HSI microcirculatory parameters to mortality and acute kidney injury due to the low incidence in the study cohort. In a future study, additional HSI measurements during physical activity might provide further insights into the pathophysiology of microcirculatory perfusion before and after TAVI. Since we used only left ventricular ejection fraction to assess cardiac function in this study, future studies investigating microcirculatory imaging should include an objective measurement of cardiac output to correlate the macro- and microcirculation.

Another limitation of our study is that we did not adjust for further periprocedural parameters that may have influenced kidney function such as the amount of contrast agent applied ahead and during TAVI. Nevertheless, a previous study by Goebel et al. surprisingly showed that the amount of contrast agent applied intra-procedurally did not affect the risk of acute kidney injury (27).

In conclusion, there were several interrelations found hinting that the hemodynamic coherence between the macrocirculation and the microcirculation are outcome-relevant and we were able to demonstrate a correlation between microcirculatory imaging parameters and kidney function.

This is the first study to show that microcirculatory imaging might have a potential clinical applicability for AKI in patients undergoing TAVI.

As risk management of interventional cardiovascular procedures becomes increasingly important to further enhance patient outcome, there is great potential in bedside evaluation of the microcirculation. In prospective clinical studies applying hyperspectral imaging to identify at-risk patients, nephroprotective strategies could be applied such as euvolemia at the time of TAVI, contrast-sparing techniques, discontinuation of concomitant nephrotoxins such as aminoglycosides, non-steroidal anti-inflammatory agents or vancomycin (18). Further, approaches to guide hemodynamic therapy based on skin perfusion quality and water content could potentially reduce periinterventional complications.

## Conclusion

Hyperspectral imaging was able to detect early changes in tissue oxygenation, perfusion quality, and tissue water content in response to TAVI. The HSI based microcirculatory parameters were related to the relevant clinical outcomes kidney function and longterm physical capacity after TAVI. Since an objective method for bedside microcirculatory monitoring has not yet been incorporated into clinical practice, non-invasive HSI may be a highly valuable technique for assessing the effects of cardiovascular interventions and hemodynamic therapy on the microcirculation.

## Data availability statement

The raw data supporting the conclusions of this article will be made available by the authors, without undue reservation.

## Ethics statement

The studies involving human participants were reviewed and approved by the Institutional Ethics Committee of the Medical Faculty of Heidelberg University (reference number S-128/2021). The patients/participants provided their written informed consent to participate in this study.

## Author contributions

MD, CN, RA, MW, NF, CL, FL, and DF contributed to the conception of the study. MD, AA, TH, FL, and DF contributed to the design of the study. MD, AA, A-CK, AS-F, FN, FL, and DF acquired the data, and wrote the first draft of the manuscript. MD, TH, and DF conducted the statistical analysis and primary interpreted the data. MD and TH created the figures. CN, A-CK, AS-F, RA, FN, MW, NF, CL, and FL critically revised the manuscript for important intellectual content. All authors read and approved the final version of the manuscript.

## Supplementary material

Supplementary Table 1 Median and Interquartile Range of Hyperspectral Imaging at T1, T2, and T3 in Patients undergoing TAVI; HSI parameters were taken at three different timepoints: before valve replacement (T1), after valve replacement (T2) and three days after valve replacement (T3). Tissue oxygenation (StO_2_), tissue hemoglobin index (THI), near-infrared perfusion index (NPI), tissue water index (TWI) values range from 0 to 100. StO_2_ is given in % and THI, NPI and TWI are index values in arbitrary units.
