# Supplementary material for: Microcirculatory tissue oxygenation correlates with kidney function after transcatheter aortic valve implantation–Results from a prospective observational study
Source: Front Cardiovasc Med. 2023 Feb 14;10:1108256. doi: 10.3389/fcvm.2023.1108256 (PMC9971913; doi:10.3389/fcvm.2023.1108256)
Supplement: Supplementary Table 1 — Median and Interquartile Range of Hyperspectral Imaging at T1, T2, and T3 in Patients undergoing TAVI; HSI parameters were taken at three different timepoints: before valve replacement (T1), after valve replacement (T2) and three days after valve replacement (T3). Tissue oxygenation (StO2), tissue hemoglobin index (THI), near-infrared perfusion index (NPI), tissue water index (TWI) values range from 0 to 100. StO2 is given in % and THI, NPI and TWI are index values in arbitrary units. [file Table_1.PDF]

	T1	T2	T3
S_tO_2 % Hand	64 (58;70)	51 (45;62)	62 (56;66)
S_tO_2 % Finger	74.875 (63.75;79.25)	63.875 (51.25;71.5)	72.875 (66.5;76.25)
NIR Hand	62 (56;66)	54 (47;61)	60 (55;66)
NIR Finger	72.5 (64.5;79)	58.875 (51.75;70)	69 (63.25;75.75)
THI Hand	28 (21;33)	29 (25;33)	25 (21;32)
THI Finger	46.75 (38.5;54.5)	50.875 (44.5;57)	48.5 (41.25;52.75)
TWI Hand	58 (55;60)	57 (51;62)	61.5 (55;67)
TWI Finger	51.25 (45;55.25)	53.375 (46.75;58.5)	55.25 (49.75;60.5)
